# Structural plasticity of the selectivity filter in a nonselective ion channel

**DOI:** 10.1107/S205225252100213X

**Published:** 2021-04-02

**Authors:** Raktim N. Roy, Kitty Hendriks, Wojciech Kopec, Saeid Abdolvand, Kevin L. Weiss, Bert L. de Groot, Adam Lange, Han Sun, Leighton Coates

**Affiliations:** a Neutron Scattering Division, Oak Ridge National Laboratory, 1 Bethel Valley Road, Oak Ridge, TN 37831, USA; b Department of Molecular Biophysics, Leibniz-Forschungsinstitut für Molekulare Pharmakologie, 13125 Berlin, Germany; c Computational Biomolecular Dynamics Group, Max Planck Institute for Biophysical Chemistry, 37077 Göttingen, Germany; d Structural Chemistry and Computational Biophysics, Leibniz-Forschungsinstitut für Molekulare Pharma­kologie, 13125 Berlin, Germany; e Institut für Biologie, Humboldt-Universität zu Berlin, Berlin, Germany; f Second Target Station, Oak Ridge National Laboratory, 1 Bethel Valley Road, Oak Ridge, TN 37831, USA

**Keywords:** ion channels, membrane proteins, X-ray crystallography, solid-state NMR, molecular dynamics

## Abstract

The sodium potassium ion channel (NaK) is a nonselective ion channel that conducts both sodium and potassium across the cellular membrane. A new crystallographic structure of NaK reveals conformational differences in the residues that make up the selectivity filter between the four subunits that form the ion channel and the inner helix of the ion channel. The crystallographic structure also identifies a side-entry, ion-conduction pathway for Na^+^ permeation that is unique to NaK. NMR studies and molecular dynamics simulations confirmed the dynamical nature of the top part of the selectivity filter and the inner helix in NaK as also observed in the crystal structure. Taken together, these results indicate that the structural plasticity of the selectivity filter combined with the dynamics of the inner helix of NaK are vital for the efficient conduction of different ions through the non-selective ion channel of NaK.

## Abstract 2

The X-ray structure of a nonselective sodium potassium ion channel reveals structural asymmetry in the selectivity filter of an integral membrane protein responsible for ion permeation. NMR studies showed the dynamical nature of the protein structure in the membrane, and molecular dynamics suggest different degrees of conformational plasticity are required for conducting potassium and sodium.

## 1. Introduction   

Both potassium and sodium ions are highly abundant in biological systems, playing key roles in the electrical activity of cells. The sodium potassium ion channel (NaK) is a non­selective, monovalent cation channel that conducts both sodium and potassium ions equally efficiently (Shi *et al.*, 2006 ▸). In contrast, potassium ion channels selectively conduct potassium ions over sodium ions by a factor of 1000:1 (Hille, 2001 ▸). This high selectivity for potassium ions is achieved by a highly conserved amino acid sequence (Thr-Val-Gly-Tyr-Gly) that forms a narrow pore within the ion channel called the selectivity filter (SF). In the SF of potassium ion channels, four identical binding sites are formed from the backbone carbon­yls and a threonine hydroxyl group (Doyle *et al.*, 1998 ▸). The high conductance of potassium ion channels can be explained by either the multi-ion ‘knock-on’ mechanism (Kratochvil *et al.*, 2016 ▸; Jensen *et al.*, 2010 ▸), or the more recently proposed ‘direct knock-on’ mechanism (Köpfer *et al.*, 2014 ▸; Langan *et al.*, 2018 ▸; Öster *et al.*, 2019 ▸). The SF of NaK (Thr-Val-Gly-Asp-Gly) differs by a single amino acid residue (Asp66) to the one found in potassium ion channels, yet NaK conducts both sodium and potassium ions. The mechanism by which NaK is able to conduct both sodium and potassium ions is currently under active debate (Sauer *et al.*, 2013 ▸; Roux, 2017 ▸; Nimigean & Allen, 2011 ▸). NaK has a similar architecture to that of potassium ion channels such as KcsA (Doyle *et al.*, 1998 ▸); potassium ion channels possess four discrete ion binding sites, each with a seemingly identical chemical environ­ment. NaK also possesses four discrete binding sites, the first binding site is an external site found at the top of the SF, the second binding site occurs within a water-filled cavity often called the vestibule, the third and fourth binding sites occur at the bottom of the SF. The chemical environment at the second ion binding site is defined by ordered water molecules within the vestibule and is thus dynamical, while the third and fourth ion binding sites in the SF are conserved between NaK and potassium ion channels (Alam & Jiang, 2009*a* ▸,*b* ▸). Mutagenesis studies on NaK have shown that the mutations Asp66Tyr and Asn68Asp radically alter the conformation of the first two binding sites in the SF of NaK, generating a potassium-selective ion channel mutant named NaK2K (Derebe *et al.*, 2011 ▸). The ion selectivity of the NaK2K mutant shows that a Tyr residue within the SF precludes the permeation of sodium ions.

The structure of the full-length NaK from *Bacillus cereus* was first solved to a resolution of 2.40 Å in the space group *C*222_1_ (Shi *et al.*, 2006 ▸). In this structure, two of the four polypeptide chains that make up a complete ion channel are found within the asymmetric unit of the crystal. In a later study, it was found that the deletion of the first nineteen amino acid residues removed the interfacial helix of the protein, altering the conformation of the NaK channel and placing it in an ‘open’ form as the inner helices twist around a flexible glycine residue (Gly87). This deletion mutant is often named NaKΔ19. To date, all the reported crystal structures (Alam & Jiang, 2009*a* ▸; Liu & Gonen, 2018 ▸) of NaKΔ19 have crystallized in space group *I*4 in which only one of the four polypeptides that make up a complete ion channel is found within the asymmetric unit. The other three polypeptides are generated from the symmetry of the crystal, meaning that all four subunits are identical. This has yielded an incomplete picture of how the complete biological subunit of the NaK ion channel operates. NaK crystals in this space group (*I*4) are also highly prone to either perfect or partial merohedral twining (Parsons, 2003 ▸). In this form of twinning, Bragg reflections perfectly overlap each other, giving a seemingly normal looking diffraction pattern. Only upon careful analysis of the Bragg reflection intensity profiles can merohedral twinning be detected, and the amount of twinning estimated and refined (Parsons, 2003 ▸). Several excellent methods and tests (Padilla & Yeates, 2003 ▸) have been developed by the crystallographic community to detect this and other sorts of twinning. However, it is not unknown for this sort of twinning to go undetected during refinement (Yeates & Fam, 1999 ▸). Here, we were able to crystallize NaKΔ19 in the original space group of the full-length NaK, namely *C*222_1_. In this space group, two polypeptides or subunits are present within the asymmetric unit allowing differences between subunits to be observed. An added benefit of the space group *C*222_1_ is that merohedral twinning is not possible, removing additional complications from structural refinement. These crystals diffracted to a resolution of 1.53 Å producing the highest resolution structure of NaKΔ19 to date. Previous structures of NaKΔ19 typically have lower resolutions between 1.80 and 2.40 Å (Shi *et al.*, 2006 ▸; Alam & Jiang, 2009*a* ▸).

Recently, we used a combination of solid-state NMR and MD simulations, revealing conformational plasticity of the SF as the key for conducting both Na^+^ and K^+^ efficiently (Shi *et al.*, 2018 ▸). While we found K^+^ to permeate through the SF in the fourfold symmetric crystallographic conformation, an asymmetric structure in different subunits is required for Na^+^ conduction. MD simulations further suggested a previously undiscovered binding site for Na^+^ outside the SF, which is caused by a peptide flip of Asp66 and named, by us, a ‘side-entry’ binding site. The determined asymmetric X-ray structure from the present study does not only corroborate the dynamic and asymmetric nature of the SF in NaK, but also nicely confirms the presence of the side-entry binding site necessary for Na^+^ conduction.

## 2. Materials and methods   

### 2.1. X-ray crystallography protein expression and purification   

A gene encoding a truncated form of NaK from *Bacillus cereus* m1550 without the 19 amino acid residues of the N-terminal M0 helix was purchased in the pD421 expression vector from ATUM (Newark, CA). After transformation and plating of *E. coli* BL21 cells on LB agar plates, colonies were resuspended in liquid LB medium, grown at 37°C to an A_600_ of approximately 0.8, and induced with 0.5 m*M* IPTG for 18 h at 25°C. Cells were then pelleted by centrifugation. For every gram (wet weight) of cells, 5 ml of lysis buffer (50 m*M* Tris pH 7.8, 150 m*M* KCl) containing SIGMAFAST protease inhibitor (Millipore Sigma) and 1 mg ml^−1^ of lysozyme was added. Cells were resuspended by slow stirring at room temperature for 30 min and further lysed by sonication. Cell debris was removed by centrifugation at 10 000 relative centrifugal force (r.c.f.). NaK was then solubilized by incubation of the supernatant with 40 m*M* Sol-grade *n*-decyl-β-d-malto­pyran­oside (DM) at room temperature for 2 h. Additional debris was removed from the lysate by centrifugation at 21 000 r.c.f. for 30 min. The protein was then purified on a TALON metal affinity resin using buffers containing 4 m*M* DM. Fractions containing NaK were pooled, and the 6×His-Tag was removed by adding one unit of thrombin per milligram of NaK and incubating at room temperature for 16 h. NaK was then concentrated via ultrafiltration using a 30 kDa MWCO concentrator and further purified on a Superdex 200 Increase 10/300GL column using 20 m*M* Tris–HCl pH 7.8, 100 m*M* KCl and 4 m*M* Anagrade DM.

### 2.2. Protein crystallization   

A solution of NaK protein solution was concentrated via ultrafiltration to 14 mg ml^−1^ using a 50 kDa MWCO concentrator. The crystals used for data collection were grown in sitting drops prepared by mixing equal volumes of protein solution in buffer (20 m*M* Tris–HCl pH 8.0, 50 m*M* KCl 50 m*M* NaCl and 4 m*M* Anagrade DM) with well solution (72.5% MPD, 50 m*M* KCl and 100 m*M* HEPES pH 7.5). Crystals were flash-frozen in liquid nitro­gen without any additional cryoprotectant.

### 2.3. 100 K X-ray data collection processing and refinement   

Two sets of X-ray diffraction data were collected at 100 K over a range of 100° (0.5° steps) from the same crystal using a Dectris PILATUS3 X 6M detector at the Advanced Photon Source (APS) on the ID19 beamline SBC-CAT to 1.53 Å resolution. All data were processed and reduced using *XDS* (Kabsch, 2010 ▸) and *XSCALE*. Molecular replacement was used for phasing the structure via the *Molrep* program from the *CCP4* suite (Winn *et al.*, 2011 ▸). Model building and refinement were conducted using *Coot* (Emsley *et al.*, 2010 ▸) and *phenix.refine* from the *Phenix* suite (Adams *et al.*, 2010 ▸), while *PyMOL* (Yuan *et al.*, 2016 ▸) was used to make all the figures within the manuscript. The data collection and refinement statistics are given above in Table 1 ▸.

### 2.4. ssNMR materials and methods   

#### 2.4.1. Protein purification   

NaKΔ19 was expressed in *E. coli* NEB express *I* ^Q^ (New England Biolabs). Minimal medium (M9) was supplemented with ^13^C glucose (4 g l^−1^; 552151, Sigma–Aldrich) and ^15^N ammonium chloride (1 g l^−1^; 299251, Sigma–Aldrich). At an OD_600_ of 0.8, the culture was induced with 0.4 m*M* IPTG (iso­propyl-β-d-1-thio­galacto­pyran­oside) and grown overnight at 25°C. Cells were harvested and resuspended with lysis buffer [50 m*M* Tris (pH 7.5), 100 m*M* NaCl, 1 m*M* MgCl] and protease inhibitor cocktail (11836170001, Sigma–Aldrich). The sample was lysed using a microfluidizer (LM10, Microfluidics) with five cycles at a working pressure of 15 000 p.s.i. The membrane fraction was isolated by ultracentrifugation at 150 000 r.c.f. for 2 h at 4°C and solubilized in solubilization buffer [50 m*M* Tris (pH 7.5), 100 m*M* NaCl and 40 m*M* DM (*n*-decyl-β-maltoside; GLYCON Biochemicals)] at room temperature for 3 h. The protein was batch-purified by immobilized metal affinity chromatography using TALON Superflow beads (GE Healthcare Life Sciences). Protein concentrations were determined using Bradford reagent supplemented with α-cyclo­dextrin (5 mg ml^−1^; AppliChem). The protein sample was mixed with *E. coli* total lipid extract (100500, Avanti Polar Lipids) in a ratio of 2:1 (*w*:*w* protein to lipid) for 1 h at 4°C. Reconstitution into liposomes was performed by dialysis at 4°C for 8 d against 20 m*M* Tris (pH 8.0), 50 m*M* NaCl and 50 m*M* KCl with 5× buffer exchange and a 100× dilution factor. The proteoliposome pellet was collected by ultracentrifugation at 300 000 r.c.f. for 4 h at 4°C. The sample was filled into a 3.2 mm magic angle spinning (MAS) rotor (Bruker BioSpin) and used for measurements, sample *A* with mixed-ion conditions.

The sample was subsequently washed from 50 m*M* NaCl and 50 m*M* KCl conditions to only 50 m*M* KCl. For this, the sample was removed from the rotor and resuspended in 1 ml buffer with 20 m*M* Tris (pH 8.0) and 50 m*M* KCl. The buffer was refreshed twice, after ultracentrifugation for 30 min at 150 000 r.c.f. and 4°C, and the total incubation time was 14 d. After this time, the sample was ultracentrifuged at 300 000 r.c.f. and 4°C for 2 h and again filled into a 3.2 mm MAS rotor. Sample *B* contained only K^+^ buffer conditions.

#### 2.4.2. ssNMR spectroscopy   

Solid-state NMR spectra were recorded on a 16.4 T wide-bore spectrometer (700 MHz, Bruker BioSpin) equipped with a 3.2 mm triple-resonance Efree MAS probe. Spectra were recorded at 11 kHz MAS and referenced with external DSS (4,4-di­methyl-4-sila­pentane-1-sulfonic sodium salt). The sample temperature was kept constant at approximately 10°C as measured by the temperature-dependent position of the water resonance. The 90° pulse radio frequency amplitudes were 83.3, 50 and 35.7 kHz for proton, carbon and nitro­gen, respectively. High-power proton decoupling was performed using the SPINAL-64 sequence during the evolution and detection periods. The samples were characterized using 2D ^15^N_i_–^13^Cα_i_ (NCA) and ^13^C–^13^C correlation spectra. The carbon–carbon correlated proton-driven spin diffusion (PDSD) spectra were collected with a mixing time of 20 ms. The NCA experiment employed a SPECIFIC CP step with a contact time of 2.8 ms for sample *A* and 3 ms for sample *B*.

### 2.5. MD simulations   

MD simulations were performed using the *GROMACS* software package (version 2019; Van Der Spoel *et al.*, 2005 ▸). In the simulations, an F92A mutant of the crystal structure of NaK (PDB entry 6V8Y) was embedded in a patch of a 1-palmitoyl-2-oleoyl-*sn*-glycero-3-phosphocholine (POPC) membrane and solvated in water with K^+^ or Na^+^ and Cl^−^ ions, corresponding to a salt concentration of 600 m*M*. Instead of a WT-NaK channel, the F92A mutant was used in all simulations in order to increase the current as an experimentally increased ion flux was reported for this mutant (Alam & Jiang, 2009*a* ▸,*b* ▸). All titratable residues of the protein were proton­ated according to their standard protonation state at pH 7. Simulations were performed with both AMBER99sb and CHARMM36m (Adams *et al.*, 2010 ▸) force fields using the TIP3P water model (Wu *et al.*, 2014 ▸).

For the simulations with the AMBER99sb force field, Berger’s parameters for lipids (Berger *et al.*, 1997 ▸) and Joung’s parameters for ions (Joung & Cheatham, 2008 ▸) were employed in both the equilibrium and the production runs. Aliphatic hydrogen atoms were treated with the virtual sites approach (Feenstra *et al.*, 1999 ▸), allowing for a 4 fs time step for integration. To equilibrate the systems, a short 10 ns simulation was performed by position-restraining all heavy atoms of the NaK channel with a force constant of 1000 kJ mol^−1^ nm^−2^ to the starting structure. The system was further equilibrated for 20 ns by releasing the position restraints. For simulations with the CHARMM36m force field, *GROMACS* inputs and parameters were provided by the CHARMM-GUI webserver (Jo *et al.*, 2008 ▸, 2009 ▸; Wu *et al.*, 2014 ▸; Lee *et al.*, 2016 ▸). The system was equilibrated in six steps using default scripts provided by the CHARMM-GUI webserver. A time step of 2 fs was used in the simulations of 2.875 ns for equilibration.

After the double-bilayer setup was built, the system was further equilibrated without position restraints for 5 ns in the Amber setup and 10 ns in the CHARMM setup. For all production simulations with Amber99sb and CHARMM36m force fields, short-range electrostatic interactions were calculated with a cutoff of 1.0 nm, whereas long-range electrostatic interactions were treated by the particle mesh Ewald method (Darden *et al.*, 1993 ▸; Essmann *et al.*, 1995 ▸). The cutoff for van der Waals interactions was set to 1.0 nm, while long-range dispersion corrections for energy and pressure were applied. Simulations were performed at a constant temperature of 310 K with a velocity rescaling thermostat (Bussi *et al.*, 2007 ▸). The pressure was kept constant at 1 bar by means of a semi-isotropic Brendensen barostat (Berendsen *et al.*, 1984 ▸). All bonds were constrained with the LINCS algorithm (Hess *et al.*, 1997 ▸). During the production simulations, an ion imbalance (Na^+^ or K^+^) of 2 and 4 was maintained between two compartments in the computational electrophysiology setup (Kutzner *et al.*, 2011 ▸, 2016 ▸) as implemented in the *GROMACS* package. The resulting transmembrane potential was calculated using the *GROMACS* tool *gmx potential*. A permeation event was counted when an ion passed through the entire SF. For simulations at larger degrees of opening of inner helices (Table S1 of the supporting information), we used distance restraints between CA atoms of the A92 residue, similar to our recent work (Kopec *et al.*, 2019 ▸). The distances between four CA atoms were increased from the one present in the crystal structure of the NaK2K channel (PDB entry 3ouf; Derebe *et al.*, 2011 ▸) by either 0.1 or 0.2 nm, with a force constant of 1000 kJ mol^−1^ nm^−2^.

## 3. Results and discussion   

**Figure 1:**
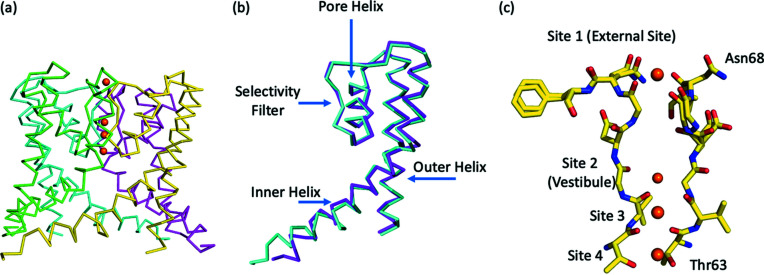
(*a*) NaK ion channel composed of four subunits, each of which is formed from a separate polypeptide. Each of the four polypeptides or subunits are shown in a different color with the ions within the SF shown as orange spheres. (*b*) Overlay of the alpha carbon traces of the two polypeptide chains in our NaKΔ19 structure. The A polypeptide chain is shown in magenta, and the B polypeptide chain is shown in cyan. The SF forms the ion channel pore, next to that is the pore helix, which is flanked by the long inner helix, the outer helix is located at the periphery of the ion channel. (*c*) SF of the NaK ion channel shown in stick format with ions in the SF shown as orange spheres. For clarity only two of the four subunits that make up a complete ion channel are shown.

Within the asymmetric unit of our structure is a dimer that contains two of the four polypeptides that make up the complete NaK ion channel [Fig. 1 ▸(*a*)]. We calculated the RMSD for alpha carbon atoms between the two polypeptide chains (A and B) in the dimer within our structure (1.40 Å) using the least-squares fit function (LSQ) in *Coot* (Emsley *et al.*, 2010 ▸) [Fig. 1 ▸(*b*)]. We also used *Coot* to calculate the RMSD for all atoms in the A and B polypeptide chains (1.96 Å).

**Figure 2:**
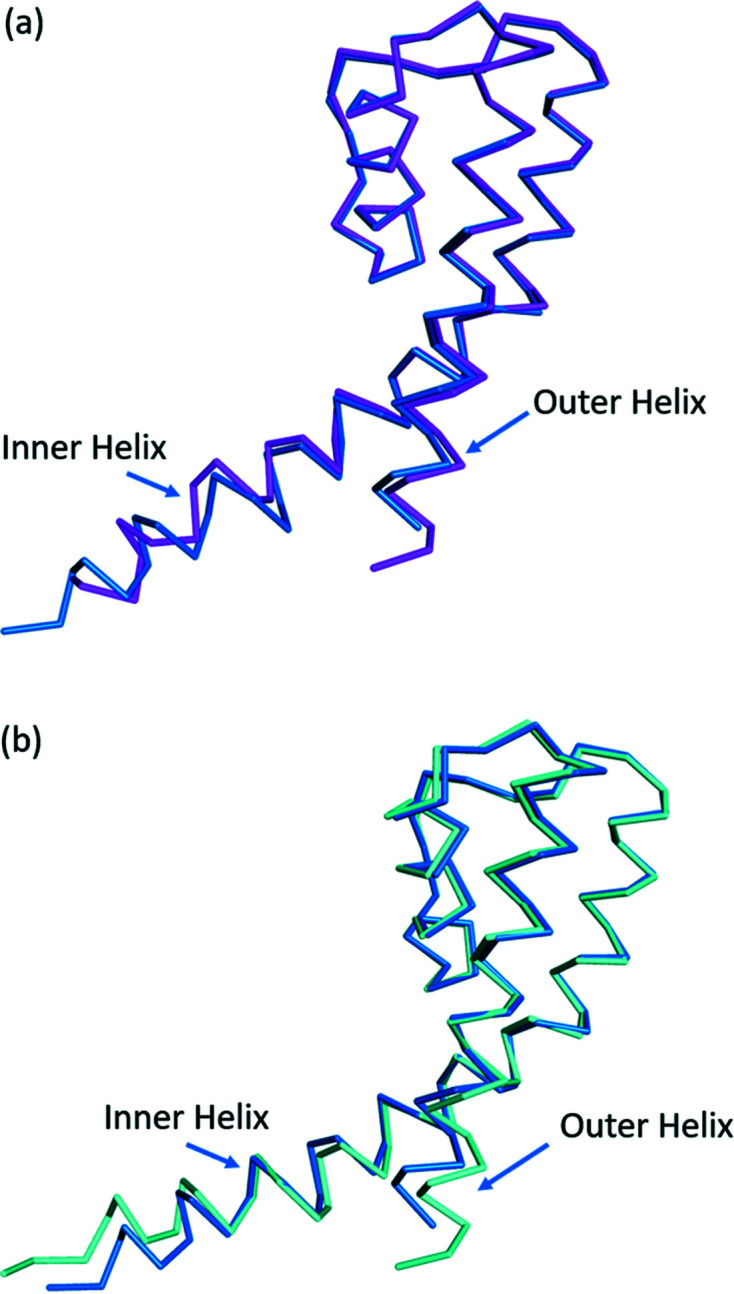
Overlay of the α-carbon traces between the two polypeptides within our NaKΔ19 structure and a previous NaKΔ19 structure in a different space group. (*a*) A polypeptide of our NaKΔ19 structure shown in magenta, and a previously refined 1.80 Å structure of NaKΔ19 (PDB entry 3e8h) shown in blue. (*b*) B polypeptide of our NaKΔ19 structure shown in cyan, and a previously refined structure of NaKΔ19 (PDB entry 3e8h) shown in blue.

As can be seen from Fig. 1 ▸, the conformation of the inner helix differs between the A and B polypeptides in the dimer within our structure. We then compared our NaKΔ19 structure with the previous crystal structure of NaKΔ19 reported by Alam & Jiang (2009*a* ▸) in space group *I*4 (PDB entry 3e8h). When comparing LSQ fits between 3e8h with the A polypeptide chain in our structure, we see an RMSD for alpha carbon atoms of 1.24 and 1.66 Å for all atoms in the structure. Comparing LSQ fits between 3e8h and the B polypeptide in our structure, the RMSD for alpha carbon atoms is 0.87 and 1.09 Å for all atoms in the entire structure. This indicates that the conformations of both the A and the B polypeptides in our structure differ from those seen in earlier work on NaKΔ19 (Fig. 2 ▸).

Several differences are visible in the conformation of the inner helix below the hinge region between our structure and previous work on NaKΔ19. A flexible glycine residue (Gly87) is found in the middle of the inner helix. It is thought to act as a gating hinge (Alam & Jiang, 2009*a* ▸; Jiang *et al.*, 2002 ▸) in NaK, and other tetrameric ion channels such as MthK, a calcium-gated potassium ion channel (Jiang *et al.*, 2002 ▸) from *Methano­bacterium thermoautotrophicum*. In the original structure of NaKΔ19 in the space group *I*4, only one subunit is present within the asymmetric unit. The other three subunits are identical, symmetry-related copies of the original subunit. The fact that the conformation of the inner helix differs between the subunits and that the conformation of the inner helix in both of our subunits differs from the original structure in space group *I*4 indicates the conformation of the inner helix is dynamic and is also influenced by crystal packing inter­actions. This is in accord with the recent solid-state NMR studies on NaKΔ19 which failed to assign residues below the gating hinge (Gly87) in the inner and outer helices due to conformational heterogeneity caused by strong structural dynamics (Shi *et al.*, 2018 ▸) in this region. The conformation of the outer helix differs between the two subunits in our structure and that of the previously solved structure.

Electrophysiology studies have been conducted on NaK and NaKΔ19 channels reconstituted into liposomes to study ion selectivity via the incorporation of radioactive ^86^Rb (Shi *et al.*, 2006 ▸) into the liposome. In these studies, a much higher flux rate was observed for NaKΔ19 that was three times higher than that of NaK. This is understandable as NaKΔ19 lacks the N-terminal M0 helix-forming residues that are thought to lock the channel in a closed form (Alam & Jiang, 2009 ▸). These studies (Shi *et al.*, 2006 ▸) suggest that the ion channel conducts K^+^ ions more effectively than Na^+^ ions and that the NaK channel is not very selective between Na^+^, K^+^ and Rb^+^.

### 3.1. Asymmetry within the selectivity filter   

**Figure 3:**
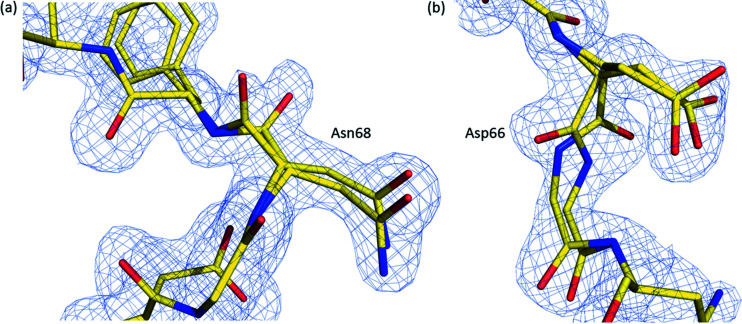
Two key residues involved in selective ion permeation within the homodimer present in our structure are in dual conformations. (*a*) In the A polypeptide of our homodimer, Asn68 is present in two conformations, the 2*F*_o_−*F*_c_ electron density map contoured at a level of 1σ is shown as a blue mesh. (*b*) In the B polypeptide of our homodimer, Asp66 is present in two conformations, the 2*F*_o_−*F*_c_ electron density map contoured at a level of 1.3σ is shown as a blue mesh.

During refinement, it became evident that the entire Asn68 residue in the A polypeptide was present in dual conformations and that the entire Asp66 residue in the B polypeptide was also present in dual conformations. Each of the two conformations of Asn68 [Fig. 3 ▸(*a*)] in the A polypeptide has a refined occupancy of around 50%. In one conformation, the ND2 atom of the Asn68 side chain interacts with the backbone carbonyl of Asn68 on the B polypeptide (2.87 Å), whereas in the other conformation the ND2 atom interacts with the side-chain hydroxyl OG atom of Ser70 on the B polypeptide chain (3.01 Å). The conformation of the Asn68 side chain in the original NaKΔ19 structure is similar to the conformation that interacts with the backbone carbonyl of Asn68 on the B polypeptide. However, it is not a particularly close match. The backbone carbonyl of Asn68 is also present in dual conformations, although the difference is minor compared with that of Asp66 in the B polypeptide chain.

**Figure 4:**
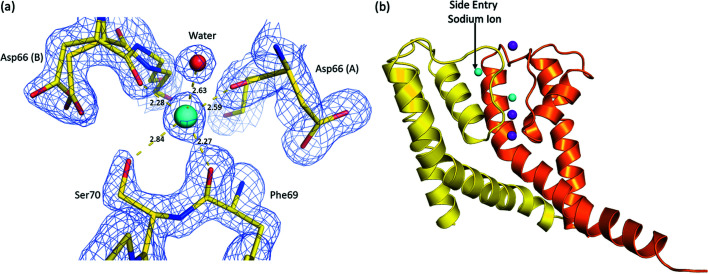
Potential side-entry sodium ion binding site found just outside of the SF. (*a*) 2*F*_o_−*F*_c_ electron density map contoured at 1σ shown as a blue mesh. Coordination distances to a putative sodium ion (cyan sphere) are given in Ångstroms. The Asp66(A) residue comes from the A polypeptide, whereas the Asp66(B) residue comes from the B polypeptide. (*b*) The position of the potential side entry sodium ion binding site is shown, the a polypeptide subunit is shown as a yellow cartoon while the a polypeptide subunit is shown as an orange cartoon. Potassium and sodium ions are shown as purple and cyan spheres, respectively.

The residue Asn68 is found at the top of the SF, where it forms a constriction point. Thus, the ability of Asn68 to alter its conformation could serve to alter the diameter of external entry to the SF. As potassium and sodium ions have different ion radii (Harding, 2002 ▸), this structural feature could allow the permeation of both sodium and potassium to enter and permeate through NaK. Each of the two conformations of Asp66 in the B polypeptide [Fig. 3 ▸(*b*)] also has a refined occupancy of around 50%. In one conformation its main chain carbonyl is parallel with the SF where it interacts with a water molecule (2.30 Å) that hydrogen bonds to one of four water molecules (2.30 Å) involved in coordinating the bound sodium ion at the second ion binding site within the vestibule of the SF. This conformation of the main-chain carbonyl of Asp66 is the one observed in previous NaKΔ19 structures (Alam & Jiang, 2009*a* ▸,*b* ▸). In the second conformation, the carbonyl of Asp66 undergoes a peptide flip and coordinates to a partially occupied putative sodium ion binding site outside of the SF (Fig. 4 ▸). This flipped carbonyl group of Asp66 is only present in one of the two polypeptides (B) within our structure. The putative sodium ion binding site (Fig. 4 ▸) is formed from the backbone carbonyl oxygen atom of Asp66 (2.28 Å), the backbone carbonyl oxygen atom of Phe69 (2.27 Å), the backbone carbonyl oxygen atom of Asp66 on a symmetry-related molecule (2.59 Å), a water molecule (2.63 Å) and the side-chain hydroxyl of Ser70 (2.84 Å). The Asp66 side chain is also present in dual conformations; one conformation interacts with the backbone amide group of Phe69 (2.67 Å) on the same polypeptide chain while the other conformation interacts with the hydroxyl of Ser70 (2.63 Å) on a symmetry-related A polypeptide chain. The conformation of the Asp66 side chain in the original NaKΔ19 structure interacts with the backbone amide group of Phe69 and has its carbonyl group pointing into the SF.

Molecular dynamics simulations have recently shown that the conduction of sodium atoms via NaK involves a side-entry ion conduction pathway (Shi *et al.*, 2018 ▸). This side entry pathway results from a flipping of the backbone carbonyl on Asp66, which is present in the B polypeptide in our structure. The observed structural plasticity within the SF with its ability to alter conformation is likely to be a critical molecular determinant for highly efficient conduction of different ions in non-selective cation channels. This role of carbonyl flipping in the SF of NaK makes sense, as main-chain carbonyl groups from a protein are the most common donor groups to sodium ions (Harding, 2002 ▸).

### 3.2. Contents of the selectivity filter   

**Figure 5:**
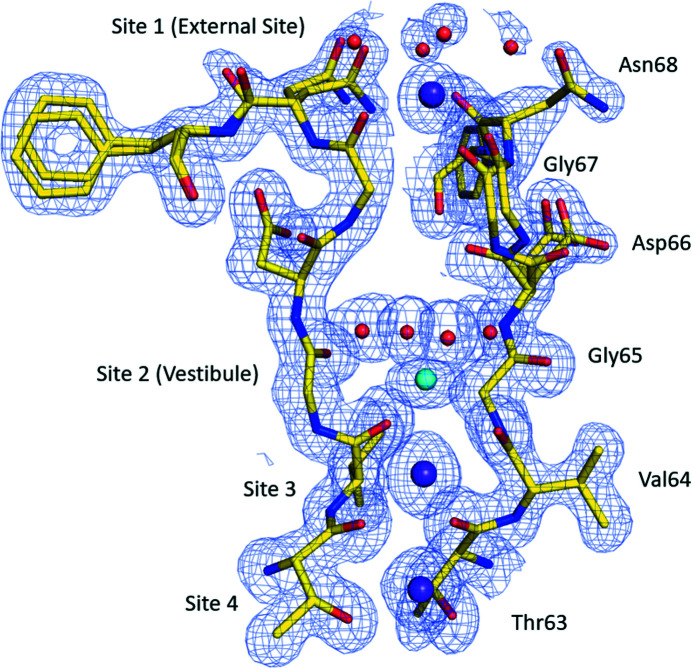
SF of the NaK ion channel. For clarity, only the two polypeptide chains found within the dimer of our structure are shown. Potassium ions are shown as purple spheres, with sodium ions shown as cyan spheres and water molecules are shown as red spheres. A 2*F*_o_−*F*_c_ electron density map contoured at 1.0σ is shown as a blue mesh.

Within our ion channel structure, a potassium ion is bound at the external site found at the top of the SF (Fig. 5 ▸). This potassium is coordinated to four backbone carbonyl oxygen atoms of Gly67 (2.60 Å) and four ordered water molecules (2.80 Å) found at the top of the ion channel. In the vestibule, a sodium ion coordinates to four water molecules (2.60 Å) and the four backbone carbonyl oxygen atoms of Val64 (3.00 Å). While in site 3, a potassium ion is coordinated to the backbone carbonyl oxygen atoms of Thr63 (2.83 Å) and Val64 (3.01 Å). Site 4 in the ion channel is also occupied by a potassium ion, which coordinates to the four backbone carbonyl oxygen atoms of Thr63 (2.89 Å) and the four hydroxyl oxygen atoms of Thr63 (2.79 Å) that line the SF. The sodium ion and water molecule structure observed within the vestibule of our NaK structure are very similar to those seen in a previous structure of a 100 m*M* Na^+^ complex (Alam & Jiang, 2009*b* ▸) of NaK (PDB entry 3e89). However, in the previous structure, the water–sodium coordination distance was an unrealistic 4.02 Å, compared with the much more favorable 2.60 Å distance (Harding, 2002 ▸) observed in our structure. The distance observed between the sodium ion and the four backbone carbonyl oxygen atoms of Val64 in this structure (PDB entry 3e89) is 2.94 Å, which is very similar to the 3.00 Å distance observed in our structure. This coordination distance is somewhat longer than might be expected for a sodium ion but may reflect a mixture of ions within the vestibule (site 2).

### 3.3. Conformational flexibility of the SF corroborated with solid-state NMR data   

**Figure 6:**
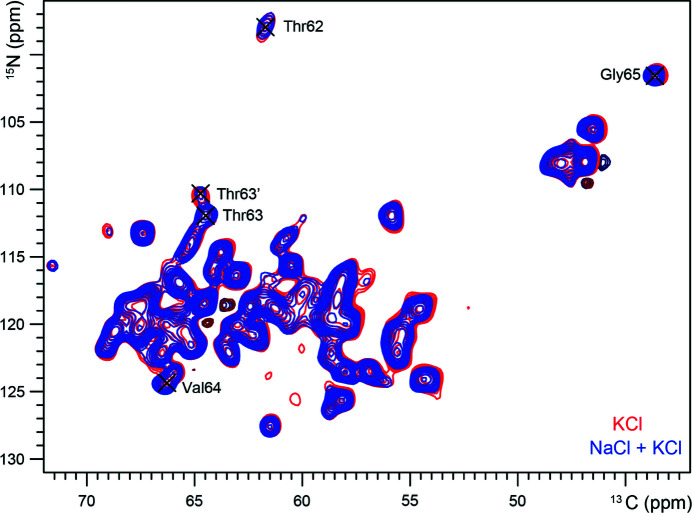
2D ^15^N–^13^Cα correlation spectra of NaKΔ19 in 50 m*M* K^+^ (positive signals in red; negative signals in maroon) and with 50 m*M* K^+^ and 50 m*M* Na^+^ (positive signals in blue; negative signals in dark blue). Indicated are the residues belonging to the lower part of the SF.

To further investigate the influence of mixed sodium and potassium conditions on NaKΔ19, we performed solid-state NMR measurements, which had previously only been performed under pure sodium or potassium conditions. We prepared uniformly [^13^C-^15^N]-labeled NaKΔ19 in proteo­liposomes with 50 m*M* potassium and 50 m*M* sodium present. The 2D ^15^N–^13^Cα correlation spectrum can be seen in Fig. 6 ▸ (in blue) with the SF residues indicated. As observed before, spectra of NaK do not display signals for residues in either the inner or outer helix below the height of the gating hinge (Gly87). Notably, residues Asp66 up to Ser70 cannot be observed either (Shi *et al.*, 2018 ▸), the absence of these peaks is likely the result of conformational flexibility. The residues in the upper part of the SF match those found in multiple conformations or varying interactions in our current crystal structure, which could explain the loss of their NMR signals.

To enable a comparison with pure potassium conditions, we changed the buffer of the sample to 50 m*M* potassium and measured it again. This procedure allows for an accurate comparison as the same sample is used for all measurements. The resulting overlay of the spectra clearly shows that the samples are virtually identical (Fig. 6 ▸). The spectra show that the overall structure of the protein is not influenced by the change to pure potassium conditions (see also Fig. S1 of the supporting information). No differences can be observed for the residues which make up the site 3 and site 4 binding sites, Thr63 and Val64. The entire lower part of the SF under potassium conditions appears to be unchanged from mixed sodium and potassium conditions.

### 3.4. Molecular dynamics based computational electro­physiology simulations suggest that larger reorganization of the selectivity filter is needed for Na^+^ conduction   

**Figure 7:**
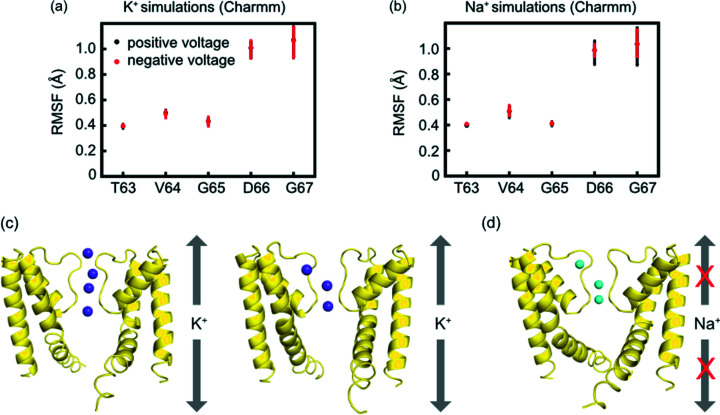
Root mean square fluctuations (RMSF) of the SF during MD simulations with (*a*) K^+^ and (*b*) Na^+^ using the CHARMM36m force field. Snapshots from the MD simulations showing (*c*) top-to-bottom and side-entry pathways of K^+^ permeation and (*d*) no Na^+^ permeation.

To investigate whether the asymmetric X-ray structure represents the conductive state of the NaK channel, we performed molecular dynamics based computational electrophysiology simulations (Kutzner *et al.*, 2011 ▸, 2016 ▸) for K^+^ and Na^+^ using the Amber99sb (Lindorff-Larsen *et al.*, 2010 ▸) and CHARMM36m (Huang & MacKerell, 2013 ▸) force fields, respectively. During the simulations, we observed significantly higher RMSFs at the top part of the SF (Asp66 and Gly67) compared with the lower ones [Thr63, Val64 and Gly65, Figs. 7(*a*) and 7(*b*)], which is in good agreement with the X-ray structure showing the same pattern of conformational flexibility.

From the MD simulations, we observed inward as well as outward K^+^ permeation using both Amber99sb and CHARMM36m force fields (Table 2 ▸). The simulated K^+^ conductance using the new asymmetric SF structure (0.8–1.3 pS, Table 2 ▸) is comparable with our previously simulated one (0.6 pS) based on the same method but starting from the fourfold symmetric structure (Shi *et al.*, 2018 ▸). Compared with the experimental conductance (Derebe *et al.*, 2011 ▸) (35 pS), the simulated conductance from the current study is about 27–44 times smaller. Nevertheless, the conductance ratio between the previously simulated MthK channel (MthK: 7.2 pS for outward K^+^) (Köpfer *et al.*, 2014 ▸) and NaK studied here is about 5–9. This agrees relatively well with the experimentally observed single-channel conductance ratio (MthK/NaK: 6.3) (Derebe *et al.*, 2011 ▸).

Interestingly, K^+^ could permeate through the SF via two different pathways [Fig. 7 ▸(*a*)]: (i) the canonical pathway, where the ion transverses through the entire SF, similar to K^+^ permeation in selective K^+^ channels like KcsA (Köpfer *et al.*, 2014 ▸); (ii) the side-entry pathway, where K^+^ binds at the side-entry binding site (the same side-entry binding site for Na^+^ determined in the current X-ray structure) before leaving the SF. This result is different from our previous simulations of NaK based on the fourfold symmetric structure, where only the canonical conduction pathway was observed during K^+^ conduction through the SF (Shi *et al.*, 2018 ▸). As previous Na^+^ permeation simulations based on the asymmetric SF also allowed side-entry permeation (Shi *et al.*, 2018 ▸), we conclude that the deformation away from the fourfold symmetric pore domain enables the side-entry conduction pathway for both K^+^ and Na^+^ ions.

In strong contrast to the K^+^ permeation, we did not observe any Na^+^ permeation from our simulations, even at high transmembrane voltages (>600 mV, Table 2 ▸). Na^+^ remained tightly bound in the planes formed by backbone carbonyls of T63 or V64 for the duration of the simulations [Fig. 7 ▸(*d*)], similar to our earlier simulations of NaK starting from the fourfold symmetric structure (Shi *et al.*, 2018 ▸). Our previous study further revealed that the Na^+^ conductive state requires an asymmetric deformation of the lower part of the SF (induced by the flipping of Thr62) which was suggested by the ssNMR data. This leads to a considerably wider SF to accommodate solvated Na^+^ ions during permeation. In the current study, the channel was crystallized when both K^+^ and Na^+^ are present in the buffer solution. The new structure reveals that K^+^ ions bind at sites 3 and 4, whereas the Na^+^ ion is found in the vestibule as well as at the side-entry binding site. This result is in line with the ssNMR data, showing that the lower part of the SF (Thr63, Val64 and Gly65) is stabilized by K^+^ at the mixed ionic condition. Therefore, we conclude that the current crystal structure with only conformational asymmetry at the top part of the SF represents only the K^+^-conducting state that enables ion permeations from two different pathways. In contrast, the Na^+^-conducting state of NaK is likely to exhibit a much larger conformational plasticity at the entire SF.

Recent simulations of the potassium-selective mutant of NaK (NaK2K) revealed that outward K^+^ permeation can be regulated by relatively minor alterations of the SF width, that is, in turn, affected by the degree of opening of inner helices (Kopec *et al.*, 2019 ▸). To probe whether the same mechanism is present in NaK, we performed additional MD simulations, imposing a predefined opening of inner helices with distance restraints (Table S1 ▸ of the supporting information). The average opening at S4 reached slightly lower levels to those observed for increased current in NaK2K (*ca* 0.855–0.860 nm) in the CHARMM force fields, suggesting that the coupling between the inner helices and the SF might be weaker in NaK. Nevertheless, we observed that the larger opening could indeed enhance outward K^+^ conduction, especially with the CHARMM force field. In contrast, we did not observe any Na^+^ ion permeation events, similarly to smaller openings sampled in unrestrained simulations (Table 2 ▸), further suggesting that the larger degree of SF reorganization is needed for Na^+^ conduction. Of interest, channels permeating ions in the inward direction consistently attained a lower level of the S4 opening than the ones permeating in the outward direction. This observation suggests that also the SF occupancy by ions, that differ for inward and outward permeation, might have an effect on the coupling.

## 4. Conclusions   

Our X-ray structure, NMR data and molecular dynamics simulations have revealed the dynamical nature of the inner helix in NaK and the different conformations of the inner helix below the gating hinge indicate the flexibility of this region. The asymmetry between polypeptides observed within the dimer in our asymmetric unit and the different conformations of Asp66 and Asn68 observed in the two polypeptides within the asymmetric subunit indicate the structural plasticity of the SF in NaK. The observed asymmetry reveals a so-far unseen sodium ion binding site adjacent to the SF that enables the side entry of ions into the ion channel. The presence of this side-entry binding site agrees with earlier MD simulations (Shi *et al.*, 2018 ▸). Thus, we propose that the asymmetry and plasticity of Asp66 within the SF helps to enable NaK to conduct both sodium and potassium with high efficiency.

The structure and corresponding structure factors have been deposited into the Protein Data Bank with the PDB entry 6v8y.

## Supplementary Material

Supplementary Table and Figure. DOI: 10.1107/S205225252100213X/mf5049sup1.pdf

PDB reference: sodium potassium ion channel NaK, 6v8y

## Figures and Tables

**Table 1:** Data collection and refinement statistics for the NaK ion channel structure

PDB entry	6v8y
Wavelength (Å)	0.97
Space group	*C*222_1_
*a*, *b*, *c* (Å)	81.72, 88.14, 49.85
α, β, γ (°)	90, 90, 90
Resolution range (Å)	38.34–1.53 (1.58–1.53)
Total No. of reflections	188414 (11639)
No. of unique reflections	26758 (2568)
Completeness (%)	97.10 (94.69)
Multiplicity	7.04 (4.53)
〈*I*/σ(*I*)〉	16.20 (1.90)
CC_1/2_	0.999 (0.678)
*R*_p.i.m._ (%)	2.23 (45.20)
*R*_factor_(%)	15.69
*R*_free_ (%)	17.77
RMSD bond lengths (Å)	0.008
RMSD bond angles (°)	0.963
Ramachandran favored (%)	99.44
Ramachandran outliers (%)	0.00

**Table 2:** Summary of MD-based computational electrophysiology simulations of the NaK channel Simulations of NaK (PDB entry 6v8y) for Na^+^ and K^+^ using different force fields (Amber: Amber99sb; CHARMM: CHARMM36) and varying transmembrane voltages.

Ion	Force field	Ion imbalance	Voltage (mV)	Time (µs)	No. of production runs	Inward permeation (number µs^−1^)	Inward conductance (number µs^−1^)	Outward permeation (number µs^−1^)	Outward conductance (number µs^−1^)
K^+^	Amber	2	390 ± 30	5	5	2 ± 1	0.8 ± 0.4	0	0
K^+^	Amber	4	600 ± 30	5	5	4 ± 3	1.1 ± 0.8	1 ± 2	0.3 ± 0.5
K^+^	CHARMM	2	380 ± 60	5	10	1 ± 1	0.4 ± 0.4	3 ± 3	1.3 ± 1.3
Na^+^	Amber	2	380 ± 60	5	5	0	0	0	0
Na^+^	Amber	4	640 ± 50	2.5	5	0	0	0	0
Na^+^	CHARMM	2	340 ± 10	5	10	0	0	0	0
