# Supplementary material for: Structural plasticity of the selectivity filter in a nonselective ion channel
Source: IUCrJ. 2021 Apr 2;8(Pt 3):421–30. doi: 10.1107/S205225252100213X (PMC8086165; doi:10.1107/S205225252100213X)
Supplement: Supplementary file 1 [file m-08-00421-sup1.pdf]

IUCrJ

Volume 8 (2021)

Supporting information for article:

Structural plasticity of the selectivity filter in a nonselective ion channel

Raktim N. Roy, Kitty Hendriks, Wojciech Kopec, Saeid Abdolvand, Kevin L. Weiss, Bert L. de Groot, Adam Lange, Han Sun and Leighton Coates

Table S1 Simulations at larger degrees of opening of inner helices of NaK, we used distance restrains placed between CA atoms of A92 residue

Opening	Opening A92 [nm]*	Opening T63 [nm]*	Ion	Force field	Voltage [mV]	Number of simulations / Total simulation time [µs]	Inward permeation [number/µs]	Outward permeation [number/µs]
0.1	ch0: 1.756 ± 0.003 ch1: 1.760 ± 0.003	ch0: 0.838 ± 0.004 ch1: 0.845 ± 0.006	K^+	Amber	370±40	5 / 2.5	2±2	0
0.1	ch0: 1.761 ± 0.006 ch1: 1.770 ± 0.004	ch0: 0.843 ± 0.004 ch1: 0.839 ± 0.005	K^+	Amber	620±20	5 / 2.5	4±1	0
0.2	ch0: 1.868 ± 0.002 ch1: 1.874 ± 0.002	ch0: 0.844 ± 0.007 ch1: 0.864 ± 0.01	K^+	Amber	570±50	5 / 2.5	6±6	1±1
0.1	ch0: 1.748 ± 0.004 ch1: 1.749 ± 0.003	ch0: 0.858 ± 0.003 ch1: 0.867 ± 0.005	Na^+	Amber	410±30	5 / 2.5	0	0
0.1	ch0: 1.745 ± 0.005 ch1: 1.743 ± 0.003	ch0: 0.857 ± 0.003 ch1: 0.860 ± 0.002	Na^+	Amber	610±40	5 / 2.5	0	0
0.2	ch0: 1.834 ± 0.001	ch0: 0.831 ± 0.001	K^+	Charmm	360±40	3 / 1.5	3±2	14±7

	ch1: 1.843 ± 0.005	ch1: 0.837 ± 0.002						
0.1	ch0: 1.734 ± 0.002 ch1: 1.733 ± 0.001	ch0: 0.845 ± 0.001 ch1: 0.848 ± 0.001	Na^+	Charmm	330±10	5 / 2.5	0	0
0.1	ch0: 1.734 ± 0.001 ch1: 1.736 ± 0.001	ch0: 0.844 ± 0.001 ch1: 0.852 ± 0.001	Na^+	Charmm	540±10	5 / 2.5	0	0
0.2	ch0: 1.834 ± 0.002 ch1: 1.836 ± 0.001	ch0: 0.848 ± 0.001 ch01: 0.856 ± 0.001	Na^+	Charmm	540±20	5 / 2.5	0	0

*ch0 - channel with inward permeation, ch1 - channel with outward permeation

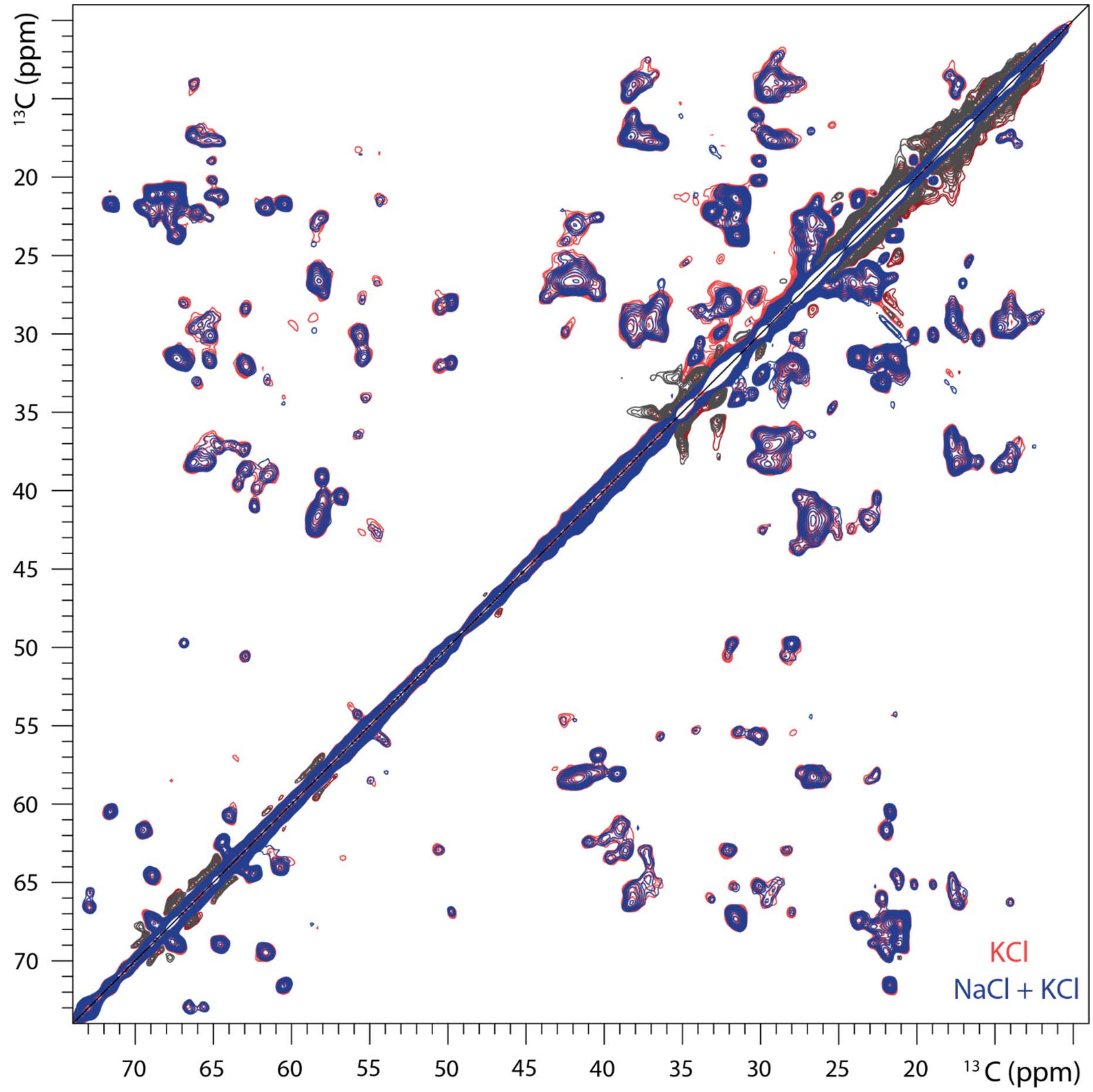

Figure S1 2D ^{13}C-^{13}C proton-driven spin diffusion (PDSD) correlation spectra of NaKΔ19 in 50mM K^{+} (positive signals in red; negative signals in maroon) and with 50 mM K^{+} and 50 mM Na^{+} (positive signals in blue; negative signals in grey).
